# Coverage of Large-Scale Food Fortification of Edible Oil, Wheat Flour, and Maize Flour Varies Greatly by Vehicle and Country but Is Consistently Lower among the Most Vulnerable: Results from Coverage Surveys in 8 Countries^1^,^2^,^3^

**DOI:** 10.3945/jn.116.245753

**Published:** 2017-04-12

**Authors:** Grant J Aaron, Valerie M Friesen, Svenja Jungjohann, Greg S Garrett, Lynnette M Neufeld, Mark Myatt

**Affiliations:** 4 Global Alliance for Improved Nutrition, Geneva, Switzerland; and; 5 Brixton Health, Llawryglyn, Wales, United Kingdom

**Keywords:** large-scale food fortification, wheat flour, maize flour, edible oil, program coverage

## Abstract

**Background:** Large-scale food fortification (LSFF) of commonly consumed food vehicles is widely implemented in low- and middle-income countries. Many programs have monitoring information gaps and most countries fail to assess program coverage.

**Objective:** The aim of this work was to present LSFF coverage survey findings (overall and in vulnerable populations) from 18 programs (7 wheat flour, 4 maize flour, and 7 edible oil programs) conducted in 8 countries between 2013 and 2015.

**Methods:** A Fortification Assessment Coverage Toolkit (FACT) was developed to standardize the assessments. Three indicators were used to assess the relations between coverage and vulnerability: *1*) poverty, *2*) poor dietary diversity, and *3*) rural residence. Three measures of coverage were assessed: *1*) consumption of the vehicle, *2*) consumption of a fortifiable vehicle, and *3*) consumption of a fortified vehicle. Individual program performance was assessed based on the following: *1*) achieving overall coverage ≥50%, 2) achieving coverage of ≥75% in ≥1 vulnerable group, and *3*) achieving equity in coverage for ≥1 vulnerable group.

**Results:** Coverage varied widely by food vehicle and country. Only 2 of the 18 LSFF programs assessed met all 3 program performance criteria. The 2 main program bottlenecks were a poor choice of vehicle and failure to fortify a fortifiable vehicle (i.e., absence of fortification).

**Conclusions:** The results highlight the importance of sound program design and routine monitoring and evaluation. There is strong evidence of the impact and cost-effectiveness of LSFF; however, impact can only be achieved when the necessary activities and processes during program design and implementation are followed. The FACT approach fills an important gap in the availability of standardized tools. The LSFF programs assessed here need to be re-evaluated to determine whether to further invest in the programs, whether other vehicles are appropriate, and whether other approaches are needed.

## Introduction

Large-scale food fortification (LSFF)^6^, the focus of the current article, relies on commonly consumed food vehicles (i.e., staple foods) to deliver micronutrients to as much of the general population as possible while also trying to include a large proportion of members of vulnerable population groups who would stand to benefit most from additional micronutrients (1). This approach to delivering micronutrients has a long history of success to address inadequate dietary intake of essential nutrients in higher-resource countries (2–5), and is increasingly used in low- and middle-income countries to address a range of micronutrient deficiencies (1, 6, 7). LSFF programs generally fall into 2 categories: *1*) mandatory, whereby all producers of branded and packaged fortifiable foods should fortify the selected vehicles according to national legislation standards; and *2*) voluntary, whereby producers may choose to fortify of their own accord, usually according to a national voluntary fortification standard. The former should achieve higher coverage levels at the population level, assuming legislation standards are followed (i.e., producers are compliant).

Despite being widely practiced, many LSFF programs in lower-resource settings have not been able to demonstrate impact (8). This is due to failures to generate, access, or apply data during program design (i.e., for the selection of appropriate vehicles and fortificants) and implementation (i.e., routine program monitoring and evaluation activities for continuous program enhancements). Tools to assist fortification program managers throughout the program cycle are essential to ensure that programs are designed and implemented appropriately.

The WHO has published a general guidance document on fortification practices (1) and an updated consensus statement on recommended fortification levels (9). To facilitate the collection and analysis of the data required to select appropriate food vehicles and fortification levels, the main tool that is available is the Fortification Rapid Assessment Tool (FRAT), which was developed in the late 1990s (10). FRAT surveys have been successfully implemented in several countries before starting programs, particularly in Africa (11). One limitation of the FRAT approach is that although the method emphasizes assessing women and children, it fails to explicitly assess vulnerability, relying instead on overall consumption patterns of these population groups to select appropriate vehicles (10, 11). Programs that have not carried out intake assessments have generally relied on more indirect assessments, such as estimating per capita consumption based on vehicle production estimates to select vehicles for fortification or using data from household expenditure and consumption surveys. There are limitations with such methods, as described elsewhere (12, 13). During the program implementation phase, there are fewer standardized tools available to facilitate program monitoring. One tool that is available is the Fortification Monitoring and Surveillance tool, which was designed to help track the effectiveness of a flour fortification program over time (14). The Fortification Monitoring and Surveillance tool relies largely on available monitoring and surveillance data, and provides little guidance on how such data should be collected. Detailed monitoring manuals have been developed to encourage standardized regulatory and commercial monitoring practices for some vehicles, notably salt, edible oil, and wheat flour (15–17). Regulatory and commercial monitoring practices vary widely by program and context, and are generally dependent on whether enforcement is carried out by government stakeholders. There is far less guidance available to facilitate household-level monitoring practices, and, unsurprisingly, many programs in low- and middle-income countries with ongoing LSFF programs have failed to assess program coverage of the fortifiable or fortified vehicle (6). Without such information, program managers have a very limited understanding of the degree to which an LSFF program can address or is addressing need, and whether, e.g., alternative vehicles or interventions are required.

In 2013, the Global Alliance for Improved Nutrition developed and operationalized the Fortification Assessment Coverage Toolkit (FACT) to support coverage assessments in both population-based (e.g., LSFF) and targeted (e.g., infant and young child feeding) fortification programs (18). The toolkit was designed to assess program coverage and utilization, as well as to facilitate the program feedback loop by identifying bottlenecks and barriers to coverage that could and should be addressed during implementation. The aim of this article was to review and summarize coverage findings from FACT surveys conducted in 8 countries between 2013 and 2015. A total of 18 fortification programs were assessed (7 wheat flour, 4 maize flour, and 7 edible oil programs). The overall aim of this work was to assess the coverage of these programs (i.e., what program implementation has achieved), as well as to determine whether vulnerable or at-risk population groups benefited from the respective programs.

## Methods

### 

#### Fortification program characteristics.

The fortification program activities in countries in which FACT surveys were implemented are shown in **Table 1**. Wheat flour programs were implemented in 7 countries (Côte d’Ivoire, India, Nigeria, Senegal, South Africa, Tanzania, and Uganda). Maize flour programs were implemented in 4 countries (Nigeria, South Africa, Tanzania, and Uganda). Edible oil programs were implemented in 7 countries (Bangladesh, Côte d’Ivoire, India, Nigeria, Senegal, Tanzania, and Uganda). At the time of the surveys, fortification of wheat flour was voluntary in one country (India). Oil fortification was voluntary in 2 countries (Bangladesh and India). For all other food vehicles in each respective country, mandatory legislation to fortify the food vehicles was in place at the time the surveys were conducted.

**TABLE 1:** Summary of edible oil, maize flour, and wheat flour fortification program activities in countries in which coverage surveys were implemented^1^

Variable	Bangladesh	Côte d’Ivoire (Abidjan)	India (Rajasthan)	Nigeria	Senegal	South Africa	Tanzania^2^	Uganda
Edible oil								
Start date^3^	2013	2007	2012	2000	2009	NA	2010	2003
Legislation^4^	Mandatory	Mandatory	Voluntary	Mandatory	Mandatory	NA	Mandatory	Mandatory
Micronutrient,^5^ ppm								
Vitamin A	15–30	8	7.5	6	20	—	16–28	20–45
Vitamin D_2_	—	—	0.05	—	—	—	—	—
Vitamin E	—	—	—	—	—	—	65–190	—
Maize flour								
Start date^3^	NA	NA	NA	2000	NA	2003	2011	2003
Legislation^4^	NA	NA	NA	Mandatory	NA	Mandatory	Mandatory^6^	Mandatory^7^
Micronutrient,^5^ ppm								
Folic acid	—	—	—	1.5	—	2.0	0.5–2.5	0.5–1.5
Iron	—	—	—	—	—	35^8^	5–25^9^	10–20^9^
Vitamin A	—	—	—	9	—	1.1	0.2–1.0	0.5–1.5
Thiamin	—	—	—	—	—	2.2	1.5.6.0	3; 2
Riboflavin	—	—	—	—	—	1.7	1.5–6.0	30; 20
Niacin	—	—	—	—	—	25	15–30	2
Pyridoxine	—	—	—	—	—	3.1	2.0–7.5	—
Vitamin B-12	—	—	—	—	—	—	0.002–0.010	0.003
Zinc	—	—	—	20	—	15	20–40	20–50
Wheat flour								
Start date^3^	NA	2007	2012	2000	2009	2003	2010	2003
Legislation^4^	NA	Mandatory	Voluntary	Mandatory	Mandatory	Mandatory^10^	Mandatory^6^	Mandatory^11^
Micronutrient,^5^ ppm								
Folic acid	—	2.6	1.3	1.5	2.25–2.75	1.26; 1.43	1–5	1–5; 1–4
Iron	—	60^12^	30^13^	40.7^8^	45^12^	30.08; 35.00^8^	30–50^9^	25–55^12^
Vitamin A	—	—	—	9	—	1.6; 1.8	0.5–3.0	1–4
Thiamin	—	2.8	—	6.2	—	1.7; 1.9	5–15	6; 4
Riboflavin	—	2.8	—	3.7	—	1.6; 1.8	2.5–9.0	3; 2
Niacin	—	36.2	—	49.5	—	20.8; 23.7	40–75	60; 40
Pyridoxine	—	3.1	—	—	—	2.3; 2.6	3–10	3
Vitamin B-12	—	0.02	0.01	—	—	—	0.005–0.025	0.007
Zinc	—	55	—	20	—	15.0; 13.2	30–50	40–60; 30–50

1 NA, not applicable; ppm, parts per million.

2 Mainland Tanzania only. Zanzibar is not included in the current legislation.

3 Year in which fortification standards were initially set but not necessarily when mandatory legislation was passed.

4 Status of national legislation at the time the survey was implemented.

5 Value is the required minimum level or range of added micronutrient at retail as per the national standard that was in effect at the time the survey was implemented.

6 Vitamin A, thiamin, riboflavin, niacin, and pyridoxine are optional.

7 Different standards exist for whole (high-extraction) and degermed (low-extraction) maize flour. When required levels are different, values are shown as whole or degermed.

8 Electrolytic iron.

9 NaFeDTA, sodium iron ethylenediaminetetraacetic acid.

10 Different standards exist for brown and white wheat flour. Required levels are shown separately as brown or white.

11 Different standards exist for whole (high-extraction) and white (low-extraction) wheat flour. When required levels are different, values are shown separately as whole or white.

12 Ferrous fumarate.

13 FeSo_4_.

#### Survey instruments.

In all surveys, the instruments collected data on household- and individual-level variables. In 5 surveys (Bangladesh, Nigeria, South Africa, Tanzania, and Uganda) households and women of reproductive age were assessed. For these surveys, data were collected on demographics; socioeconomic status; education levels within the household; housing conditions; recent infant and child mortality; water, sanitation, and hygiene practices; food security; women’s dietary diversity; and coverage and consumption of fortified food vehicles. For 3 surveys (Côte d’Ivoire, India, and Senegal) households and caregivers with children in the first 2 y of life were assessed. These surveys collected the same data as above, as well as data on child health, infant and young child feeding practices, and maternal and child anthropometric measurements. All survey modules (i.e., question and indicator sets) were taken or adapted from validated guidelines where available (19, 20).

#### Ethical clearance and survey administration procedures.

Ethical clearance to conduct the coverage surveys was obtained in each setting from a national or academic institutional review board. Consent to participate was obtained from the primary survey respondent on the basis that participation in the survey was voluntary. Oral consent was obtained in 5 countries (Côte d’Ivoire, India, Nigeria, Senegal, and Uganda), and written consent was obtained in 3 countries (Bangladesh, South Africa, and Tanzania). At least 2 attempts were made to conduct the survey at each selected household.

In all surveys, data were collected by trained interviewers under the supervision of experienced field supervisors. All interviewers and field supervisors were trained before the surveys and were supervised by dedicated technical personnel during implementation. The survey instruments were pilot-tested in each setting to ensure that the language and wording of questions were clear, and that question-skip logic and response options were appropriate to the setting. In 5 countries (Côte d’Ivoire, India, Senegal, South Africa, and Uganda) data were collected with the use of paper forms. In these surveys, data quality was ensured by interactive checking (for consistency, range, and legal values) during data entry, as well as batch checking (double-entry and validation, as well as a batch application of consistency, range, and legal value checks). In 3 countries (Bangladesh, Nigeria, and Tanzania) data were collected with the use of mobile devices by using interactive checking to ensure data quality. A description of the sampling schemes used in each coverage survey is shown in **Table 2**. All surveys were designed to be representative of the population in the areas in which the surveys took place. Nationally representative surveys were conducted in 4 countries (Bangladesh, Senegal, Tanzania, and Uganda). Statewide or provincially representative surveys were conducted in 3 countries (India, Nigeria, and South Africa). A citywide representative survey was conducted in one country (Côte d’Ivoire). Results for these surveys are presented by individual state or province surveyed when ≥1 state or province was assessed (Nigeria and South Africa).

**TABLE 2:** Summary of sampling schemes used in coverage surveys^1^

Country	Data collection period	Survey population	Sampling areas	Sampling scheme	Target household sample size, *n*
Bangladesh	January–April 2015	Households and women of reproductive age (15–49 y)	National: 3 strata (urban, rural, hard-to-reach rural areas)	First-stage sampling selected 42 PSUs/stratum by PPS	1512
				Second-stage sampling selected 12 households/PSU by random selection	
Côte d’Ivoire (Abidjan)	September 2014	Caregivers with children aged 0–23 mo	Abidjan: all 10 communes	First-stage sampling selected 9 PSUs by random selection	1170
				Second-stage sampling selected 13 households/PSU by random selection	
India (Rajasthan)	December 2013–February 2014	Caregivers with children aged 0–24 mo	Statewide spatial sample	First-stage sampling selected 252 PSUs by spatial sampling	4536
				Second-stage sampling selected 18 households/PSU by systematic selection in “ribbon” villages, EPI3^2^ in “clustered” villages, and random selection in urban blocks	
Nigeria	May–June 2015	Households and women of reproductive age (15–49 y)	2 states: Kano and Lagos	First-stage sampling selected 30 PSUs/state by simple random sampling	1860
				Second-stage selected 31 households/PSU by random selection	
Senegal	October–December 2013	Women of reproductive age (15–49 y) and their children aged 0–24 mo	National: 4 strata (urban Dakar, urban medium-size towns, and 2 rural zones) following the 2011 national micronutrient survey	First-stage sampling selected 20 PSUs/stratum by PPS	1946
				Second-stage sampling selected 20 households/PSU by random selectionRural strata were oversampled	
South Africa	May–June 2015	Households and women of reproductive age (18–49 y)	2 provinces: Gauteng and Eastern Cape	First-stage sampling selected 40 PSUs/province by PPS	1720
				Second-stage sampling selected 23 households/PSU in Gauteng province and 20 households/PSU in Eastern Cape province by random selection	
Tanzania	September–October 2015	Households and women of reproductive age (15–49 y)	National: urban and rural strata	First-stage sampling selected 29 PSUs/urban stratum and 41 PSUs/rural stratum by PPS sampling	1050
				Second-stage sampling selected 15 households/PSU by random selection	
Uganda	September 2015	Households and women of reproductive age (15–49 y)	National: urban and rural strata	First-stage sampling selected 35 PSUs/stratum by PPS sampling	1101
				Second-stage sampling selected 15 (originally) or 16 (increased because of concerns about response rates in early PSUs) households/PSU by random selection	

1 EPI, Expanded Program on Immunization; PPS, probability proportional to size; PSU, primary sampling unit.

2 EPI3 is an adaptation of the within-PSU sampling method used in EPI coverage surveys. The base EPI method selects neighboring households. The EPI3 adaptation increases the distance between sampled households by selecting every third household. The purpose of this adaptation is to reduce the loss of variance associated with the use of cluster samples and proximity sampling.

#### Indicators of risk.

In this article, we used 3 indicators of risk to assess the relations between coverage and vulnerability. The risk indicators were poverty, poor women’s dietary diversity, and rural residence. These indicators were selected on the basis that they were associated with poor micronutrient status and highlighted the more marginalized subgroups within the population (21, 22). Poverty was defined by multidimensional poverty index (23). A household was classified as being in poverty if the multidimensional poverty index score was greater than or equal to one-third. Women’s dietary diversity was defined by the women’s dietary diversity score (WDDS) (24, 25). A household was classified as having a poor WDDS if the female primary survey respondent had a WDDS below the median WDDS for the survey population. Surveys conducted in Bangladesh, Côte d’Ivoire, India, and Senegal defined the WDDS based on a set of 9 food groups (24). In mid-2014, dietary diversity guidelines were updated, and a new indicator for minimum dietary diversity for women of reproductive age was defined based on a set of 10 food groups (25). Surveys conducted in Nigeria, South Africa, Tanzania, and Uganda defined the WDDS with the use of the updated set of 10 food groups from the minimum dietary diversity for women of reproductive age indicator. Rural residence was determined by reference to the data used to draw the survey sample in each setting.

#### Indicators of coverage.

**FIGURE 1:**
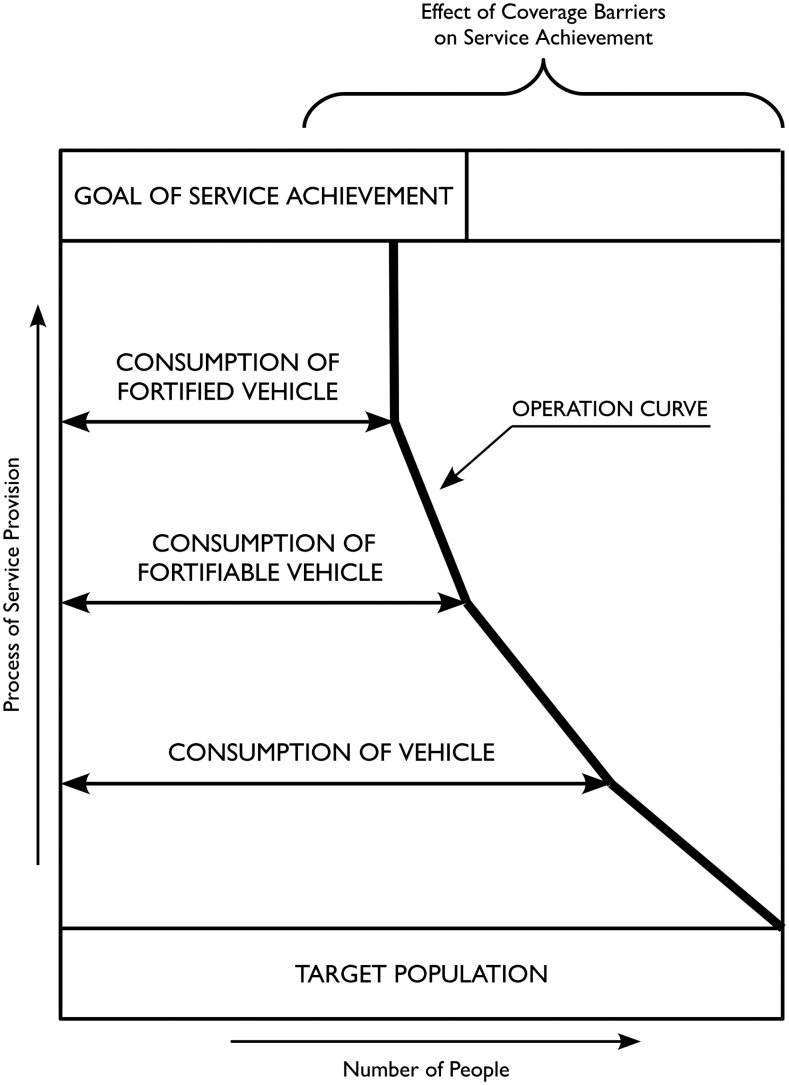
Three measures of coverage were assessed while following the Tanahashi coverage framework (26).

Three measures of coverage were assessed while following the Tanahashi coverage framework (**Figure 1**) to determine the principal program bottleneck (26). This framework relies on the identification of sequential stages through which coverage is achieved. Each stage relates to an important condition on the pathway to the provision of a service. A coverage measure is defined and measured for each stage. This is usually the proportion of the population for whom the condition is met. The key stages identified for achieving program aims (i.e., high coverage of adequately fortified food) in this article are the following: *1*) consumption of the vehicle—the household consumes the vehicle; *2*) consumption of the fortifiable vehicle—the food vehicle used by the household is processed industrially and hence is well suited to large-scale fortification; and *3*) consumption of the fortified vehicle—the vehicle used by the household is fortified. Each stage depends on all of the preceding stages being true. All stages must be true for a member of the population to be effectively covered.

If, e.g., a coverage assessment finds that 90% consume the vehicle, 20% consume the vehicle in a fortifiable form, and 18% consume the fortified vehicle, then the key program bottleneck is that the vehicle is consumed in a nonfortifiable form. For an LSFF program of wheat flour, then, this might mean that production of wheat flour is dominated by small-scale milling and that wheat flour is not a good choice of vehicle.

Three summary statistics were calculated for each measure of coverage: *1*) raw coverage (RC)—the proportion of all households that were covered (this is a measure of overall program coverage); *2*) met need (MN)—the proportion of households defined as vulnerable that were covered (this is a measure of how well the program addresses vulnerability); and *3*) coverage ratio (CR)—the ratio of the coverage in vulnerable households to the coverage in households considered to be not vulnerable.

The CR ranged between 0 and positive infinity. CR values <1 indicated that coverage favored nonvulnerable population groups. CR values >1 indicated that coverage favored vulnerable population groups. A CR of 1 indicated equitable coverage between vulnerable and nonvulnerable population groups. Further details on the RC, MN, and CR statistics are published elsewhere (19, 20).

None of the fortification programs assessed had predefined or a priori criteria for program coverage or coverage in vulnerable groups (i.e., none of the programs had a clear statement of their coverage goals). Performance for each respective program was assessed with the use of an aggregate summary of the RC, MN, and CR measures. This approach was selected to standardize analyses for crossprogram comparison and on the basis that the criteria meet reasonable program goals for an LSFF program. The criteria used in these analyses were the following: *1*) the point estimate of RC (i.e., total population coverage) should be ≥50% [this criterion indicates the minimum level of total population coverage to which an LSFF should aspire (1, 10)]; *2*) the point estimate of the MN measure should be ≥75% for ≥1 of the 3 indicators of risk that were assessed [this criterion states that an LSFF program should aspire to meet the needs of vulnerable populations (1)]; and *3*) the estimates of all CRs are not significantly <1 [this criterion states that an LSFF program should not exclude vulnerable populations (1)].

The standards associated with the criteria for the RC, MN, and CR measures can be modified or reasonable alternative criteria could be formulated. Results for the RC, MN, and CR measures are therefore presented to enable the reader to apply modified or alternative criteria. The criteria were applied for each vehicle to the highest Tanahashi coverage stage for which results were available (Figure 1). The principal program bottleneck is reported.

#### Determination of fortification status.

Fortification status for all food vehicles assessed in each country setting was determined by brand identification (i.e., by identifying the branded name of the vehicle) and by quantitative laboratory analyses (i.e., by analyzing food specimens to determine fortification levels). For quantitative analyses, food specimens were collected at the household or market level, depending on what was logistically feasible in each country setting. Specimens were shipped to reference laboratories for quantitative analyses. Households were classified as consuming a fortified or nonfortified vehicle based on the laboratory results. In cases in which a brand could not be determined in a household or a specimen was not collected, the household was classified as nonfortified in the analyses.

#### Data analyses.

Survey data were analyzed with the use of the R language for data-analysis and graphics (version 3.2.2), the R-AnalyticFlow scientific workflow system (version 3.0.1), and SAS (version 9.4). Summary statistics were calculated with the use of bootstrap estimation techniques that consisted of a set of within-primary sampling unit (PSU) survey samples that were sampled with replacement and with a probability proportion to PSU population size with the use of a roulette wheel (also known as stochastic sampling with replacement) algorithm (27). For each bootstrap replicate, a total of *m* PSUs were sampled with replacement (where *m* is the number of PSUs in the survey sample). Observations within selected PSUs were also sampled with replacement with the same within-PSU sample size that was achieved in the survey. A total of *r* = 400 bootstrap replicates were used. The resulting estimate consists of the 2.5th (lower 95% CI), 50th (point estimate), and 97.5th (upper 95% CI) percentiles of the distribution of the statistic across all replicates.

## Results

### 

#### Characteristics of survey samples.

Characteristics of the survey populations and survey response rates (defined as the proportion of the target sample size achieved) are shown in **Table 3**. Survey response rates were >85% in all countries except South Africa, which may have had selection biases because of the poor response rates in both surveyed provinces (i.e., 45.1% in Eastern Cape and 40.4% in Gauteng). The main reasons for nonresponse in these surveys were refusal from community leaders or associations, inability to access gated communities, and no one being present at home at the time of the survey.

**TABLE 3:** Characteristics of the survey populations^1^

Country	Achieved sample Size,^2^ *n*	Response rate, %	House hold size,^2^ *n*	Respondent age, y	At risk of Poverty,^3^%	Poor WDDS,^4^%	Rural,^5^%
Bangladesh	1512	100.0	4.9 (4.7, 5.2)	32.7 [15–49]	44.0 (37.5, 50.6)	NA (not used)	74.8 (68.1, 80.5)
Côte d’Ivoire (Abidjan)	1113	95.1	6.1 (5.8, 6.4)	29.0 [15–49]	21.0 (16.6, 26.3)	34.3 (31.5, 37.1)	NA (urban sample)
India (Rajasthan)	4627	102.0	6.7 (6.5, 6.8)	25.1 [16–48]	30.3 (26.9, 33.8)	23.5 (21.1, 25.6)	47.3 (45.8, 48.7)
Nigeria (Kano)	896	94.2	7.4 (7.2, 7.7)	28.3 [15–49]	68.3 (65.3, 71.4)	27.9 (24.7, 31.1)	70.4 (67.4, 73.4)
Nigeria (Lagos)	871	91.6	4.1 (4.0, 4.3)	32.0 [15–49]	8.8 (7.0, 10.7)	45.3 (41.5, 49.1)	11.9 (9.8, 14.1)
Senegal	1910	98.2	12.9 (6.2, 19.6)	28.0 [15–49]	59.9 (53.8, 66.1)	41.5 (36.1, 46.4)	66.9 (57.7, 75.2)
South Africa (Eastern Cape)	361	45.1	4.9 (4.6, 5.2)	30.0 [18–49]	33.5 (24.5, 43.6)	53.1 (46.0, 60.1)	48.4 (30.7, 66.1)
South Africa (Gauteng)	372	40.4	3.7 (3.5, 3.9)	32.7 [18–49]	19.2 (12.9, 26.9)	55.2 (48.6, 61.8)	4.0 (0.0, 14.5)
Tanzania	1036	98.7	4.4 (2.8, 6.2)	28.7 [15–49]	45.0 (37.0, 53.1)	28.4 (24.2, 32.7)	58.5 (55.4, 61.5)
Uganda	949	86.2	5.6 (5.3, 5.9)	30.1 [15–49]	63.4 (57.3, 69.6)	43.7 (38.0, 49.4)	53.6 (50.4, 56.9)

1 Values are means (95% CIs) or means [ranges]. NA, not applicable; WDDS, women’s dietary diversity score.

2 Sample size within primary sampling units sometimes exceeded quota because of *1*) exhaustive sampling in urban blocks, and *2*) extra households that were occasionally sampled from linear segments in villages.

3 Defined as multidimensional poverty index ≥0.33.

4 Defined as WDDS below median value.

5 Defined as rural place of residence.

#### Program coverage.

RC for each measure of coverage for wheat flour, maize flour, and edible oil at the household level are shown in **Table 4**. For wheat flour, only Senegal achieved RC ≥50% for consumption of the fortified vehicle (51.2%). For maize flour, only South Africa achieved RC ≥50% for consumption of the fortified vehicle (Gauteng 77.4%; Eastern Cape 86.8%). For edible oil, 3 countries (Côte d’Ivoire, Tanzania, and Uganda) achieved RC ≥50% for consumption of the fortified vehicle. For Bangladesh, RC ≥50% was achieved for consumption of fortifiable oil (88.4%), which was the highest coverage stage for which results were available for this survey. The percentage MN by risk factor (i.e., poverty, poor WDDS, and rural residence) and country for wheat flour, maize flour, and edible oil are shown in **Table 5**. Only 2 countries (Côte d’Ivoire for edible oil and South Africa for maize flour) achieved a percentage MN measure ≥75% for ≥1 risk group. One country (Senegal for wheat flour) achieved an MN measure ≥40% for ≥1 risk group. All other programs demonstrated considerably lower coverage among vulnerable population groups. CRs by risk group (i.e., poverty, poor WDDS, and rural residence) and country for wheat flour, maize flour, and edible oil are shown in **Table 6**. The trends were consistent with the results from the MN analyses. Overall program performance and program bottlenecks based on the aggregate summary of the RC, MN, and CR statistics are summarized in **Table 7**. Only 2 programs (Côte d’Ivoire for edible oil and South Africa for maize flour) met all 3 criteria. For each program, the prin-cipal bottleneck is reported for the highest level of coverage measured.

**TABLE 4:** Raw coverage of wheat flour, maize flour, and edible oil at the household level by country^1^

Country	Uses vehicle	Vehicle is fortifiable^2^	Vehicle is fortified^3^
Wheat flour			
Côte d’Ivoire (Abidjan)	54.7 (50.1, 59.6)	10.2 (7.5, 13.1)	NA^4^
India (Rajasthan)	83.2 (79.5, 86.5)	7.1 (5.6, 9.1)	6.3 (4.8, 7.9)
Nigeria (Kano)	83.9 (81.5, 86.3)	83.8 (81.4, 86.2)	22.7 (20.0, 25.5)
Nigeria (Lagos)	14.2 (11.8, 16.5)	13.8 (11.5, 16.1)	5.4 (3.8, 6.9)
Senegal	81.8 (76.2, 86.6)	81.5 (75.5, 86.4)	51.2 (44.7, 57.2)
South Africa (Eastern Cape)	25.2 (16.3, 34.1)	25.2 (16.3, 34.1)	16.3 (10.0, 23.7)
South Africa (Gauteng)	4.3 (1.8, 7.6)	4.3 (1.8, 7.6)	0.8 (0.0, 2.3)
Tanzania	51.5 (44.5, 58.5)	50.5 (43.3, 57.7)	33.1 (27.5, 38.7)
Uganda	11.2 (7.7, 14.7)	10.6 (7.6, 13.6)	8.5 (5.7, 11.4)
Maize flour			
Nigeria (Kano)	77.1 (74.4, 79.9)	11.0 (9.0, 13.1)	1.7 (0.9, 2.6)
Nigeria (Lagos)	12.2 (10.0, 14.4)	2.9 (1.8, 4.0)	0.2 (0.0, 0.5)
South Africa (Eastern Cape)	98.7 (96.5, 100.0)	98.7 (96.5, 100.0)	86.8 (80.0, 92.4)
South Africa (Gauteng)	95.6 (90.4, 98.6)	95.4 (90.3, 98.4)	77.4 (69.8, 94.9)
Tanzania	93.0 (89.7, 96.4)	36.6 (29.2, 44.0)	2.5 (1.3, 3.7)
Uganda	91.8 (87.7, 96.0)	42.4 (32.7, 52.1)	6.5 (3.3, 9.7)
Edible oil			
Bangladesh	All	88.4 (84.5, 92.3)	NA^4^
Côte d’Ivoire (Abidjan)	98.5 (97.5, 99.3)	98.0 (97.0, 99.0)	98.0 (97.0, 99.0)
India (Rajasthan)	All	89.4 (87.0, 91.8)	24.3 (21.1, 27.9)
Nigeria (Kano)	98.4 (97.6, 99.2)	35.9 (32.7, 39.1	7.6 (5.9, 9.4)
Nigeria (Lagos)	98.6 (97.8, 99.3)	22.7 (19.9, 25.5)	7.2 (5.5, 8.9)
Senegal	97.8 (96.3, 99.1)	95.0 (92.9, 96.8)	34.1 (29.1, 40.7)
Tanzania	96.2 (93.2, 99.2)	92.6 (89.0, 96.3)	53.6 (46.4, 60.8)
Uganda	89.9 (85.9, 94.0)	89.0 (84.7, 93.2)	54.4 (48.3, 60.4)

1 Values are % (95% CI). NA, not applicable.

2 The food vehicle used by the household is processed industrially.

3 The food vehicle used by the household is confirmed to be fortified by brand identification and quantitative laboratory analyses.

4 Food specimens were not collected. No fortification levels are available.

**TABLE 5:** Percentage met need by risk factor and country for wheat flour, maize flour, and edible oil coverage^1^

	Uses vehicle			Vehicle is fortifiable^2^			Vehicle is fortified^3^		
Country	Poverty^4^	Poor WDDS^5^	Rural^6^	Poverty	Poor WDDS	Rural	Poverty	Poor WDDS	Rural
Wheat flour									
Côte d’Ivoire (Abidjan)	55.4 (43.3, 65.0)	52.7 (46.1, 61.3)	NA (urban sample)	4.0 (1.0, 8.4)	6.4 (3.2, 10.0)	NA (urban sample)	NA^7^	NA^7^	NA (urban sample)
India (Rajasthan)	66.4 (59.5, 73.0)	77.4 (71.7, 83.3)	76.5 (71.3, 81.0)	5.0 (3.0, 7.5)	7.1 (4.9, 10.1)	3.0 (1.8, 4.9)	4.7 (3.0, 7.2)	5.5 (3.4, 8.2)	2.6 (1.4, 4.4)
Nigeria (Kano)	81.6 (78.3, 84.2)	84.6 (79.3, 88.9)	82.4 (79.1, 85.1)	81.4 (78.1, 84.1)	84.6 (79.3, 88.9)	82.3 (79.0, 85.0)	17.1 (14.2, 19.9)	14.5 (10.0, 19.7)	18.2 (15.0, 21.2)
Nigeria (Lagos)	10.8 (4.7, 18.1)	12.6 (8.3, 16.7)	12.1 (5.9, 19.2)	10.8 (4.7, 18.1)	12.3 (8.1, 16.1)	12.1 (5.9, 19.2)	1.6 (1.2, 5.4)	5.4 (2.9, 8.7)	5.7 (1.2, 10.5)
Senegal	78.0 (70.6, 84.1)	79.8 (72.1, 86.9)	77.7 (70.4, 83.5)	77.6 (69.5, 83.6)	79.3 (72.2, 86.5)	77.2 (68.5, 83.8)	48.0 (40.4, 55.9)	46.3 (37.9, 55.1)	49.3 (40.6, 57.3)
South Africa (Eastern Cape)	15.3 (7.0, 25.4)	15.1 (6.8, 24.7)	22.3 (11.2, 37.1)	15.1 (7.7, 24.6)	15.0 (6.3, 26.7)	22.0 (11.0, 37.4)	9.1 (3.3, 15.9)	8.1 (3.0, 16.1)	15.0 (5.1, 27.0)
South Africa (Gauteng)	15.2 (7.0, 24.7)	14.6 (7.2, 25.6)	23.7 (11.8, 38.6)	14.9 (6.1, 24.1)	15.2 (6.9, 25.1)	22.7 (12.4, 39.3)	9.2 (3.7, 15.2)	7.7 (2.8, 15.7)	15.4 (5.6, 28.2)
Tanzania	36.5 (31.7, 41.5)	45.2 (38.2, 53.1)	41.4 (37.5, 45.4)	35.4 (30.9, 40.2)	45.2 (38.2, 53.1)	40.1 (36.4, 44.4)	21.7 (17.8, 25.7)	25.9 (20.1, 31.7)	25.1 (21.6, 28.7)
Uganda	7.2 (4.8, 9.4)	10.8 (7.0, 14.7)	8.2 (6.1, 10.6)	6.2 (4.0, 8.5)	10.1 (6.6, 14.3)	7.5 (5.1, 9.9)	4.5 (2.6, 6.5)	7.9 (4.6, 11.4)	6.4 (4.2, 8.5)
Maize flour									
Nigeria (Kano)	76.4 (73.0, 79.7)	79.4 (73.7, 85.3)	75.3 (72.1, 78.2)	9.7 (7.2, 12.5)	12.5 (8.1, 17.6)	7.7 (5.6, 9.8)	1.6 (0.7, 2.7)	0.9 (0.4, 2.4)	1.0 (0.3, 1.8)
Nigeria (Lagos)	32.6 (22.6, 42.9)	11.6 (8.0, 16.2)	47.8 (39.4, 58.5)	5.9 (1.6, 11.5)	1.8 (0.4, 3.9)	4.4 (1.5, 8.6)	0	0	0
South Africa (Eastern Cape)	All	98.4 (92.7, 100.0)	All	All	98.2 (93.9, 100.0)	All	83.6 (74.6, 90.7)	84.4 (74.2, 92.7)	84.4 (74.8, 92.3)
South Africa (Gauteng)	All	98.4 (93.1, 100.0)	All	All	98.3 (93.0, 100.0)	All	83.7 (76.2, 90.0)	84.3 (75.8, 92.3)	84.4 (73.4, 92.5)
Tanzania	89.3 (85.9, 92.3)	92.8 (89.1, 95.8)	92.0 (89.6, 93.9)	23.7 (20.0, 28.0)	42.2 (36.0, 49.0)	20.6 (17.5, 24.2)	2.7 (1.3, 4.4)	3.2 (1.4, 5.8)	1.5 (0.6, 2.7)
Uganda	89.6 (86.8, 92.7)	86.7 (81.6, 91.6)	91.2 (88.7, 93.4)	35.3 (31.3, 40.0)	38.9 (32.6, 45.9)	36.4 (32.5, 40.4)	5.9 (4.0, 8.4)	5.7 (2.9, 9.1)	6.1 (4.2, 8.3)
Edible oil									
Bangladesh	82.1 (75.0, 87.5)	NA (not used)	85.3 (79.9, 89.5)	83.8 (76.0, 89.4)	NA (not used)	86.4 (80.5, 90.8)	NA^7^	NA^7^	NA^7^
Côte d’Ivoire (Abidjan)	98.2 (94.9, 100.0)	98.3 (96.7, 99.8)	NA (urban sample)	97.7 (94.4, 100.0)	98.3 (96.0, 99.7)	NA (urban sample)	97.7 (94.6, 100.0)	98.2 (96.1, 99.7)	NA (urban sample)
India (Rajasthan)	All	All	All	95.0 (92.8, 96.7)	90.7 (87.5, 93.7)	86.3 (82.9, 89.7)	19.7 (14.6, 25.4)	22.9 (16.6, 29.3)	20.5 (16.3, 25.2)
Nigeria (Kano)	98.1 (96.8, 99.0)	99.6 (98.8, 100.0)	98.5 (97.3, 99.3)	35.1 (31.0, 38.7)	39.5 (32.8, 46.9)	31.5 (27.9, 35.2)	8.3 (6.1, 10.5)	8.5 (5.1, 12.7)	7.1 (5.3, 9.2)
Nigeria (Lagos)	All	All	All	17.7 (9.9, 27.7)	26.7 (21.0, 32.0)	14.6 (8.4, 21.6)	4.8 (1.2, 9.7)	8.3 (5.1, 12.0)	4.1 (0.9, 8.2)
Senegal	97.3 (94.8, 98.8)	97.5 (95.3, 99.4)	97.2 (95.3, 99.1)	93.3 (90.1, 96.1)	94.0 (90.4, 96.9)	93.0 (90.3, 95.7)	21.5 (16.9, 26.2)	30.5 (24.4, 37.8)	23.5 (18.2, 29.9)
Tanzania	93.3 (90.2, 95.6)	94.9 (90.9, 97.6)	95.1 (93.2, 96.8)	89.4 (85.0, 92.3)	91.4 (87.1, 95.3)	90.4 (87.6, 92.7)	54.6 (50.0, 60.3)	50.6 (43.5, 56.8)	51.4 (42.7, 55.5)
Uganda	88. (85.2, 91.2)	89.4 (84.7, 93.4)	89.4 (86.5, 92.2)	86.8 (83.4, 90.1)	87.8 (82.8, 92.0)	88.3 (85.5, 91.1)	48.5 (43.4, 52.9)	52.2 (45.0, 59.2)	51.3 (46.5, 55.9)

1 Values are % (95% CI). Met need = the proportion of households defined as vulnerable that were covered. NA, not applicable; WDDS, women’s dietary diversity score.

2 The food vehicle used by the household is processed industrially.

3 The food vehicle used by the household is confirmed to be fortified by brand identification and quantitative laboratory analyses.

4 Defined as multidimensional poverty index ≥0.33.

5 Defined as WDDS below median value.

6 Defined as rural place of residence.

7 Food specimens were not collected. No fortification levels are available.

**TABLE 6:** Coverage ratio by risk factor and country for wheat flour, maize flour, and edible oil coverage^1^

	Uses vehicle			Vehicle is fortifiable^2^			Vehicle is fortified^3^		
Country	Poverty^4^	Poor WDDS^5^	Rural^6^	Poverty	Poor WDDS	Rural	Poverty	Poor WDDS	Rural
Wheat flour									
Côte d’Ivoire (Abidjan)	1.0 (0.8, 1.2)	0.9 (0.8, 1.1)	NA (urban sample)	0.3 (0.1, 0.7)	0.5 (0.3, 0.9)	NA (urban sample)	NA^7^	NA^7^	NA (urban sample)
India (Rajasthan)	0.7 (0.7, 0.8)	0.9 (0.8, 1.0)	0.8 (0.7, 0.9)	0.6 (0.3, 0.9)	1.0 (0.7, 1.4)	0.2 (0.1, 0.3)	0.7 (0.4, 1.0)	0.8 (0.5, 1.2)	0.2 (0.1, 0.3)
Nigeria (Kano)	0.6 (0.4, 0.8)	0.9 (0.9, 1.0)	1.0 (0.9, 1.1)	1.0 (0.9, 1.1)	0.8 (0.5, 1.1)	0.7 (0.5, 1.0)	1.0 (0.9, 1.0)	1.0 (1.0, 1.0)	1.0 (1.0, 1.1)
Nigeria (Lagos)	0.6 (0.4, 0.8)	0.9 (0.9, 1.0)	1.0 (0.9, 1.1)	1.0 (0.9, 1.1)	0.8 (0.5, 1.1)	0.7 (0.5, 1.0)	1.0 (0.9, 1.0)	1.0 (1.0, 1.0)	1.0 (1.0, 1.1)
Senegal	0.9 (0.8, 1.0)	1.0 (0.9, 1.0)	0.9 (0.8, 1.0)	0.9 (0.8, 1.0)	1.0 (0.9, 1.1)	0.8 (0.7, 0.9)	0.9 (0.7, 1.0)	0.9 (0.7, 1.0)	0.9 (0.7, 1.1)
South Africa (Eastern Cape)	1.1 (0.6, 1.8)	1.6 (0.7, 3.3)	1.9 (0.8, 4.1)	1.1 (0.6, 1.8)	1.6 (0.7, 3.1)	1.9 (0.8, 4.3)	1.1 (0.4, 2.5)	1.6 (0.5, 4.6)	2.4 (0.8, 6.8)
South Africa (Gauteng)	1.0 (0.5, 1.8)	1.6 (0.8, 3.3)	2.0 (0.9, 4.3)	1.0 (0.5, 1.8)	1.6 (0.8, 3.3)	2.0 (0.9, 4.1)	1.1 (0.4, 2.4)	1.5 (0.5, 4.2)	2.5 (0.8, 7.2)
Tanzania	0.6 (0.5, 0.7)	0.8 (0.7, 0.9)	0.5 (0.4, 0.6)	0.6 (0.5, 0.7)	0.8 (0.7, 0.9)	0.5 (0.4, 0.6)	0.7 (0.7, 0.8)	0.8 (0.7, 0.9)	0.7 (0.6, 0.8)
Uganda	0.9 (0.8, 0.9)	1.0 (0.9, 1.0)	0.8 (0.8, 0.9)	0.9 (0.8, 0.9)	1.0 (0.9, 1.0)	0.8 (0.7, 0.8)	0.9 (0.8, 0.9)	1.0 (0.9, 1.0)	0.9 (0.8, 0.9)
Maize flour									
Nigeria (Kano)	1.3 (1.1, 1.6)	1.0 (0.9, 1.1)	1.8 (1.5, 2.2)	1.0 (1.0, 1.1)	1.0 (1.0, 1.0)	1.0 (1.0, 1.1)	1.0 (1.0, 1.0)	1.0 (1.0, 1.0)	1.0 (1.0, 1.0)
Nigeria (Lagos)	1.0 (0.9, 1.1)	1.0 (0.9, 1.1)	1.8 (1.5, 2.2)	1.0 (1.0, 1.0)	1.0 (1.0, 1.0)	1.0 (1.0, 1.1)	0	0	1.0 (1.0, 1.0)
South Africa (Eastern Cape)	1.0 (1.0, 1.1)	1.0 (1.0, 1.1)	1.0 (1.0, 1.1)	1.0 (1.0, 1.0)	1.0 (1.0, 1.1)	1.0 (1.0, 1.1)	1.0 (0.9, 1.1)	1.0 (0.9, 1.2)	1.0 (0.9, 1.2)
South Africa (Gauteng)	1.0 (1.0, 1.1)	1.0 (1.0, 1.1)	1.0 (1.0, 1.1)	1.0 (1.0, 1.1)	1.0 (1.0, 1.1)	1.0 (1.0, 1.1)	1.0 (0.9, 1.1)	1.0 (0.9, 1.2)	1.0 (0.9, 1.2)
Tanzania	0.4 (0.2, 0.6)	0.9 (0.5, 1.7)	0.6 (0.3, 0.9)	0.7 (0.6, 0.8)	1.1 (1.0, 1.3)	0.4 (0.3, 0.5)	1.0 (1.0, 1.0)	1.0 (1.0, 1.0)	1.0 (0.9, 1.0)
Uganda	0.4 (0.2, 0.8)	0.4 (0.2, 0.7)	0.5 (0.3, 0.8)	0.7 (0.6, 0.8)	0.9 (0.8, 1.0)	0.4 (0.4, 0.5)	1.0 (1.0, 1.0)	1.0 (0.9, 1.0)	1.0 (0.9, 1.0)
Edible oil									
Bangladesh	0.4 (0.3, 0.7)	NA	0.4 (0.2, 0.8)	0.5 (0.3, 0.8)	NA	0.4 (0.2, 0.9)	NA^7^	NA^7^	NA^7^
Côte d’Ivoire (Abidjan)	1.0 (0.9, 1.1)	1.0 (0.9, 1.1)	NA (urban sample)	1.0 (0.9, 1.1)	1.0 (0.9, 11)	NA (urban sample)	1.0 (0.9, 1.1)	1.0 (0.9, 1.1)	NA (urban sample)
India (Rajasthan)	All	All	All	1.1 (1.0, 1.2)	1.0 (0.9, 1.1)	0.9 (0.8, 1.0)	0.8 (0.6, 1.0)	0.9 (0.7, 1.2)	0.6 (0.5, 0.8)
Nigeria (Kano)	All	0.6 (0.2, 1.9)	1.1 (0.3, 3.5)	0.9 (0.8, 1.1)	1.1 (1.0, 1.2)	0.9 (0.8, 1.0)	1.0 (0.9, 1.0)	1.0 (1.0, 1.1)	0.9 (0.9, 1.0)
Nigeria (Lagos)	All	0.6 (0.2, 1.9)	1.1 (0.3, 3.5)	0.9 (0.8, 1.1)	1.1 (1.0, 1.2)	0.9 (0.8, 1.0)	1.0 (0.9, 1.0)	1.0 (1.0, 1.1)	0.9 (0.9, 1.0)
Senegal	1.0 (0.9, 1.1)	1.0 (0.9, 1.1)	1.1 (0.9, 1.1)	1.0 (0.9, 1.1)	1.0 (0.9, 1.1)	0.9 (0.8, 1.0)	0.4 (0.3, 0.5)	0.8 (0.7, 1.0)	0.4 (0.3, 0.6)
Tanzania	0.2 (0.1, 0.4)	0.5 (0.2, 1.2)	0.3 (0.1, 0.6)	0.4 (0.3, 0.7)	0.7 (0.4, 1.4)	0.3 (0.1, 0.5)	1.0 (0.9, 1.2)	0.9 (0.8, 1.1)	0.9 (0.7, 1.0)
Uganda	0.6 (0.4, 1.0)	0.7 (0.4, 1.2)	0.7 (0.4, 1.0)	0.5 (0.3, 0.9)	0.7 (0.4, 1.1)	0.6 (0.4, 1.0)	0.7 (0.6, 0.8)	0.8 (0.7, 1.0)	0.6 (0.5, 0.7)

1 Values are % (95% CI). Coverage ratio = the ratio of the coverage in vulnerable households to the coverage in households considered to be not vulnerable. NA, not applicable; WDDS, women’s dietary diversity score.

2 The food vehicle used by the household is processed industrially.

3 The food vehicle used by the household is confirmed to be fortified by brand identification and quantitative laboratory analyses.

4 Defined as multidimensional poverty index ≥0.33.

5 Defined as women’s dietary diversity score below median value.

6 Defined as rural place of residence.

7 Food specimens were not collected. No fortification levels are available.

**TABLE 7:** Overall program performance and program bottlenecks for all surveyed wheat flour, maize flour, and edible oil fortification programs

			Criteria^1^			
Country	Region	Program	Raw coverage ≥50%	Met need,^2^ ≥75%	Coverage ratios,^3^ ≥1	Main program bottleneck (lowest coverage level)^4^
Bangladesh	Nationwide	Edible oil	●	●	○	Favors nonvulnerable groups
Côte d’Ivoire	Abidjan	Wheat flour	○	○	○	Bulk of vehicle is not fortifiable
		Edible oil	●	●	●	All criteria met
India	Rajasthan	Wheat flour	○	○	○	Bulk of vehicle is not fortifiable
		Edible oil	○	○	○	Bulk of vehicle is not fortified
Nigeria	Kano	Wheat flour	○	○	●	Bulk of vehicle is not fortified
		Maize flour	○	○	●	Bulk of vehicle is not fortifiable
		Edible oil	○	○	●	Bulk of vehicle is not fortifiable
Nigeria	Lagos	Wheat flour	○	○	●	Vehicle is not a staple
		Maize flour	○	○	●	Vehicle is not a staple
		Edible oil	○	○	●	Bulk of vehicle is not fortifiable
Senegal	Nationwide	Wheat flour	●	○	●	Favors nonvulnerable groups
		Edible oil	○	○	○	Bulk of vehicle is not fortified
South Africa	Eastern Cape	Wheat flour	○	○	●	Vehicle is not a staple
		Maize flour	●	●	●	All criteria met
South Africa	Gauteng	Wheat flour	○	○	●	Vehicle is not a staple
		Maize flour	●	●	●	All criteria met
Tanzania	Nationwide	Wheat flour	○	○	○	Bulk of vehicle is not fortified
		Maize flour	○	○	●	Bulk of vehicle is not fortifiable
		Edible oil	●	○	●	Favors nonvulnerable groups
Uganda	Nationwide	Wheat flour	○	○	●	Vehicle is not a staple
		Maize flour	○	○	●	Bulk of vehicle is not fortifiable
		Edible oil	●	○	○	Favors nonvulnerable groups

1 Solid dot indicates that criterion was met; based on consumption of the fortified vehicle for all with the exception of Bangladesh (oil) and Côte d’Ivoire (wheat flour), where consumption of the fortifiable vehicle was used because it was the highest level of coverage available.

2 Met need (i.e., the proportion of households defined as vulnerable that were covered) for ≥1 risk-group assessed (i.e., poverty, poor women’s dietary diversity score, or rural) is ≥75%.

3 Coverage ratio (i.e., the ratio of the coverage in vulnerable households to the coverage in households considered to be not vulnerable) for ≥1 risk-group assessed (i.e., poverty, poor women’s dietary diversity score, or rural) is ≥1.

4 “Fortifiable” refers to a food vehicle that is processed industrially; “fortified” refers to a food vehicle that is confirmed to be fortified by brand identification and quantitative laboratory analyses.

## Discussion

The FACT project fills an important void in the availability of simple, cost-effective tools that fortification programs can use to assess and diagnose program coverage. Results from the analyses highlight the importance of adequate program design and appropriate monitoring activities to ensure program success.

Only 2 of the 18 LSFF programs assessed met all program performance criteria used in the current analyses. This finding is consistent even if lower thresholds are considered for the program performance assessments. For programs with low RC, the results also indicate that coverage was not concentrated in vulnerable population groups (i.e., the MN measure was low). The main reasons for programs failing to meet the criteria were a poor choice of vehicle (i.e., the chosen vehicle was either not a staple or the bulk of the vehicle consumed was not fortifiable) and failure to fortify a fortifiable vehicle. These 2 reasons alone account for the principal bottlenecks in the 16 programs that did not meet the 3 performance criteria used in the analyses. Poor selection of a food vehicle is a failure of program planning and design. Because LSFF is not intended to change population dietary patterns, there is nothing that can be done during program implementation to increase program coverage in such instances. Failure to fortify a fortifiable vehicle may be a problem of program design (e.g., inability to include all large-scale producers in the program or absence of sufficient consolidation and centralization of production, processing, and distribution) or a problem of compliance or enforcement of fortification. Failing to cover vulnerable population groups may be a problem of access, affordability, or the fact that these at-risk groups do not consume the respective fortified food vehicles. Further assessments of these programs are required to determine whether the existing programs need strengthening, whether other food vehicles should be considered, and whether other interventions to deliver micronutrients are required.

The edible oil program in Côte d’Ivoire met the 3 program performance criteria used in the current analyses. For cost and logistical reasons, the assessment was only conducted in the capital city of Abidjan; therefore, conclusions about the rest of the country cannot be drawn from the current work. Further coverage assessments in rural and other urban areas outside of Abidjan would be needed to fully assess equity of fortification coverage in this country. South Africa’s maize flour fortification program met the program performance criteria used in these analyses in the 2 regions surveyed. These regions were selected for surveying because they are the 2 provinces with the highest population density and represent the most diverse areas of the country (28). Even though there may have been selection biases because of the poor response rates from these surveys, it is still likely that this program is performing well. South Africa has one of the most advanced economies in sub-Saharan Africa, and it is possible that the level of industrial consolidation, compliance, and government enforcement is more favorable than that in other countries in the region.

Planning of effective LSFF programs needs to be informed by detailed investigations of patterns of production, distribution, and consumption, and requires the selection of vehicles with the potential for high coverage in the population. Without this due diligence, programs rely largely on chance to achieve impact. The capital-intensive startup phase of these programs means that this is a gamble made with high stakes, as we have reported in other contexts (20). The main program bottlenecks responsible for many of the programs failing to meet the criteria used here for a good LSFF program could and should have been identified before the program started. For example, FRAT surveys conducted before these programs started would have revealed whether they were unlikely to achieve high overall coverage and therefore population level impact. Implementation of LSFF programs requires considerable and ongoing monitoring and evaluation. Effective monitoring and evaluation, particularly regulatory monitoring of the fortification process, is likely to have been lacking in some of the programs in which failure to fortify was the main program bottleneck.

Results from these analyses also highlight the importance of having multiple strategies to address micronutrient needs in the population. LSFF programs by design are not intended to be a panacea for micronutrient malnutrition in the population, and complementary strategies are needed to address specific population groups whose needs may be higher or who for various reasons may not access these fortified staples (1). Many countries do have comprehensive nutrition strategies that include targeted interventions (i.e., supplementation, home fortification, and complementary foods, among others); others include free or subsidized fortified products as part of social protection programs as a means to overcome barriers of access for the poor. For any intervention modality, sound program design, careful implementation, and routine monitoring to identify and correct implementation bottlenecks in a timely fashion are essential.

The principal strengths of the FACT project include the following: *1*) the development of a standardized toolkit to assess program coverage; *2*) a peer-review process that reviewed and refined the research approach; *3*) the use of standardized and validated indicators to assess vulnerability; and *4*) the use of program coverage assessments that were conducted in the overall population and in vulnerable subpopulation groups. As for limitations, household coverage estimates do not capture foods purchased and consumed outside of the households, such as snacks and restaurant meals. This may result in underestimating the potential coverage of fortification interventions in the population. This article reports on estimates that are common to and available from all surveys. Although not presented in this article, most of the surveys also assessed individual-level consumption of foods made with the use of the respective vehicles, which generated information on individual-level coverage and consumption (29). A second limitation is that staple foods fall into the category of fast-moving consumer goods. Repackaging of food vehicles into unbranded packaging was common across all countries. This issue likely resulted in an underestimation of coverage of fortified foods. A third limitation is that the risk factors used in these analyses do not capture all potential vulnerable populations. Definitively, biochemical and full dietary assessments may be preferred, but such assessments are expensive and logistically complex for programs to undertake in routine monitoring and evaluation assessments. One approach that could be considered would be similar to what Cameroon did before starting its fortification program. A biochemical assessment was conducted alongside a FRAT survey before starting the program (30). Such an approach lends itself to confirming which risk factors are associated with biochemical deficiencies. These risk factors could then be assessed subsequently in routine program monitoring, and evaluation activities could be conducted during the program implementation period.

Several observations have been consistent across surveys. The issue of repacking food vehicles at the market level needs to be addressed in future work. This will require linking market- and household-level monitoring activities to better understand fortification practices. Market-level assessments were conducted in some of the countries (Bangladesh, Côte d’Ivoire, and Senegal). Further development of the FACT methods should systematically include or be linked to market-level assessments. Assessing fortification status is challenging to conduct in the field. Quantitative analyses were used to determine the fortification levels of food vehicles, but are costly and time-consuming, and fortificants and micronutrients are subject to degradation over time because of storage conditions and length of time from collection to analysis. Having field-friendly tools to determine the presence of fortificants for all major food vehicles (similar to the rapid test kit that exists for testing iodine in salt and the qualitative iron spot test for determining the presence of iron in flour) need to be developed. Mobile photometers are available that can measure multiple micronutrients in flour (31) and oil matrices quantitatively (32, 33), but the approach still needs refinement to accelerate and improve accurate measurement in field settings.

## Conclusions

The FACT project successfully developed and operationalized a program-ready tool for carrying out fortification coverage assessments of LSFF programs. The results identified 2 major areas that programs need to focus on: *1*) the selection of appropriate food vehicles before programs are started, and *2*) routine monitoring of the fortification process to ensure that fortification occurs at the desired level. Where vehicles were chosen that have little potential for population impact, the use of funding to support such programs should be reconsidered. The second issue can and should be improved during the course of the program.

LSFF has been demonstrated to be a highly cost-effective intervention strategy to address micronutrient needs in the population (overall and in vulnerable groups); however, this can only be achieved when the necessary activities and processes during program design and implementation are followed. A number of the programs that were assessed have high potential for impact based on the consumption of fortifiable vehicles, a potential that can only be achieved with substantially improved compliance with fortification (26). For other programs in which nonstaple food vehicles are fortified or coverage of a fortifiable food vehicle is low, governments and industry may wish to reconsider the value of continued investment. The FACT method, if linked with routine monitoring of programs (particularly monitoring of the adequate fortification of the food vehicle), could facilitate the generation of the information required to ensure that such program improvements can be made in a timely manner.

## Footnotes

6 Abbreviations used: CR, coverage ratio; FACT, Fortification Assessment Coverage Toolkit; FRAT, Fortification Rapid Assessment Tool; LSFF, large-scale food fortification; MN, met need; PSU, primary sampling unit; RC, raw coverage; WDDS, women’s dietary diversity score.
